# Chronic exposures to fungicide pyrimethanil: multi-organ effects on Italian tree frog (*Hyla intermedia*)

**DOI:** 10.1038/s41598-017-07367-6

**Published:** 2017-07-31

**Authors:** Ilaria Bernabò, Antonello Guardia, Rachele Macirella, Sandro Tripepi, Elvira Brunelli

**Affiliations:** Department of Biology, Ecology and Earth Science, University of Calabria, Via P. Bucci 4/B, 87036 Rende, Cosenza Italy

## Abstract

Amphibian habitats are easily contaminated by several pollutants, and in agricultural landscapes the likely exposure scenario is represented by pesticides. Many of these substances are known or suspected to act as endocrine disrupting chemicals (EDCs). The goal of the present study was to assess the effects of pyrimethanil, a common-used but also overlooked fungicide, on liver, kidney and gonadal differentiation of *Hyla intermedia*. Through a multi-organ evaluation, we demonstrated that a long term exposure to two environmentally relevant concentrations of pyrimethanil (5 and 50 µg/L) elicits a range of toxic responses. First we showed that pyrimethanil induces underdevelopment of ovaries and interferes with normal sexual differentiation, thus revealing the endocrine disruption potential of this fungicide. Moreover we revealed that all considered organs are seriously affected by this fungicide and both necrosis and apoptosis contribute to the histological response. This is the first report on the effects of pyrimethanil on gonads, liver and kidney histology of a non-model species and it demonstrates that the hazardous properties of this fungicide can result from several pathological processes affecting different key compartments of amphibian.

## Introduction

The scientific literature provides substantial evidences that environmental pollution contribute to the worldwide decline of amphibian populations^1, 2^. In fact, due to their highly permeable integument, and their complex life cycle, amphibians are particularly vulnerable to the action of contaminants, both in aquatic and terrestrial habitats^1, 3, 4^. Amphibians can therefore be used in ecotoxicological studies as early indicators of environmental quality^5, 6^.

Aquatic habitats, in which amphibians live, breed and develop, are easily contaminated by a range of pollutants, and in agricultural landscapes the likely exposure scenario is represented by pesticides^1, 7^. Many of these substances have been shown to exert their adverse effects through modulation and/or disruption of endocrine functions, and are known as endocrine disrupting chemicals (EDCs)^2, 8–11^. Given the crucial role of the endocrine system in the maintenance of numerous biological, physiological and behavioural functions, damage in any part of this complex system can lead to serious disease or death.

A number of laboratory-based studies demonstrated that in amphibians, a perturbation of the endocrine system has the potential to dramatically affect all biological processes, including growth, development, gonadal differentiation, hormone levels and liver function^2, 9, 11–15^. There is also good evidence that amphibian populations, living in agricultural areas, have been affected by many endocrine-related disorders that can be linked to endocrine disrupting potential of pesticides (e.g. skewed sex ratio, greater incidences of gonadal anomalies, male and female reproductive dysgenesis, altered secondary sex characteristics, sex steroid and thyroid hormone disruption)^16–18^.

Over the past few years, fungicides use has significantly increased, especially in Europe, and as results of their repeated applications in agricultural practices they occur more frequently and/or in higher concentrations than other agrochemicals in all environmental compartments^19–23^ and references therein. In particular, fungicides are the most frequently detected agrochemicals in amphibian habitats and tissues^24, 25^. Available data indicate that fungicides, at environmentally relevant concentrations, can induce several harmful effects on amphibians, such as increased mortality and deformity^1, 26^, decreased and/or increased growth rate and development^13, 15, 26^, alteration of behaviour^27^, immunosuppression and lipid peroxidation^28, 29^. Most if not all of those harmful effects could be related to endocrine disturbance and a variety of fungicides are known or suspected to act as EDCs^8–11, 15^.

Pyrimethanil, one of the most environmentally detected fungicides in European agricultural regions, is an anilinopyrimidine fungicide extensively used in vineyards, but also on other fruits and vegetables^20–22, 30^. In surface waters nearby agricultural land, field monitoring studies reported that pyrimethanil concentrations range between 0.06 and 90 μg/L^21, 22, 30, 31^. Despite this, very limited information exist on the toxicity of pyrimethanil in aquatic vertebrates (summarized by Araújo and collaborators)^32^.

We recently demonstrated that a chronic exposure to pyrimethanil causes detrimental effects on survival, development, metamorphic traits, and body form in *H*. *intermedia* ^26^. However, in evaluating the effects of a toxicant in amphibians it is important to consider also lagged effects that may not become evident until metamorphosis^2, 12^.

On this basis, here we focused on the effects induced by an exposure to pyrimethanil during the whole developmental period in *H*. *intermedia* juveniles.

Several studies in mammalian and fish models, both *in vitro* and *in vivo*, suggested that pyrimethanil may influence the biosynthesis of sexual hormones and/or interact with sexual hormone receptor thus acting as EDC^10, 33^. Given the fact that EDCs are responsible of many adverse reproductive outcomes in developing amphibians, we first evaluated gonads histology in order to identify putative effects on sex ratio and gonadal differentiation. EDCs may also act through broader mechanisms/pathways than firstly recognized, exerting different effects in a tissue specific manner^14^, therefore we also analysed morphological alterations in two organs highly susceptible to xenobiotic toxicity.

The kidney is an important site of injury after chemical exposure, due to their involvement in a number of interrelated functions (i.e. maintenance of internal water, ion, and acid-base balance, selective reabsorption and secretion, and excretion of nitrogenous and other waste products of metabolism)^34, 35^. The liver has long been considered the major target organ for most chemicals, including EDCs, in consequence of its essential functional features (i.e. maintaining of the metabolic homeostasis including protein synthesis, storage metabolites, detoxification)^14, 34, 35^.

To our knowledge no previous studies have investigated the effects of pyrimethanil on the selected organs in amphibians.

By using a morphological approach we evaluated the differential response of three important target organs thus furnishing a comprehensive overview of pyrimethanil effects and toxicity. The individual recognition of animals exposed to the fungicide allowed us to analyse the results also in the light of our previous finding on development and deformity.

## Results

### Control

#### Gonadal differentiation and histology

We first evaluated the pattern of both ovarian and testicular differentiation for *H*. *intermedia* under basal condition (see supplementary materials).

**Figure 1:**
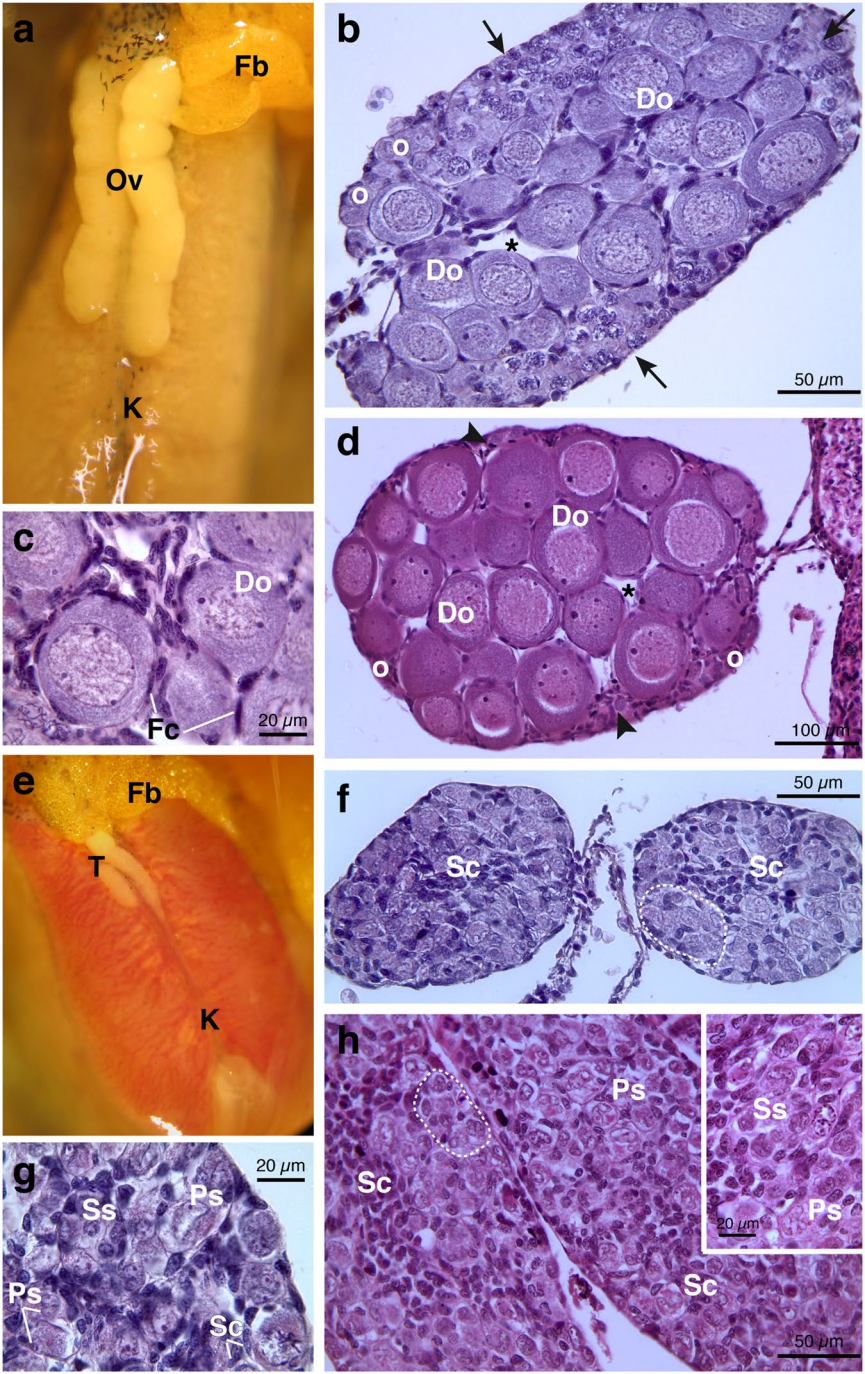
Representative photomicrographs and histological sections of ovaries and testes from *Hyla intermedia* juveniles under basal condition. (**a**) Ovaries and (**e**) testes in ventral view associated with the kidney; the fat bodies are present at the left side. (**b**,**c**) Cross-sections showing typical ovarian tissue of an ovary at stage VIII: the ovarian cavity is filled with growing diplotene oocytes; note in the cortex proliferating areas of oogonia and nests of leptotene-pachytene meiocytes (arrows). (**c**) Magnification of diplotene oocytes surrounded by follicular cells. (**d**) Cross-section of an ovary at stage IX filled by numerous and large growing diplotene oocytes; the proliferating germ cells are restricted at periphery (arrowheads). (**f**) Cross-section of testes showing proliferating spermatogonia into developing seminiferous tubules (marked by dotted lines); somatic cell are flattened and darkly stained. (**g**) At higher magnification it is possible to distinguish primary and secondary spermatogonia. (**h**) Longitudinal sections of testicular tissue showing developing seminiferous tubules. (Ov = ovaries; T = testes; K = kidney, Fb = fat body; Do = diplotene oocytes; Fc = follicular cells; o = oogonia; *=ovarian cavity; Sc = somatic cells; Ps = primary spermatogonia; Ss = secondary spermatogonia). All sections are H&E.

In *H*. *intermedia* one week after metamorphosis (age: 11–12 weeks), gonads were sexually differentiated and testes or ovaries were clearly distinguishable. Under the stereomicroscope, the differentiated ovaries appeared as paired large, long sacs with an evident external lobulation; the left gonad was slightly bigger than the right one (Fig. 1a). From histological point of view two stages of ovarian maturation were distinguishable in females: the majority (56%) of ovaries, categorized as ovary development stages VIII *sensu* ^36^, was characterized by large discontinuous areas of proliferation containing oogonia and/or nests of leptotene-pachytene meiocytes situated in the external part of the cortex, whereas numerous diplotene oocytes in primary growth (previtellogenesis) occupied ovarian lumen (Fig. 1b and Table 1). With further enlargement diplotene oocytes appeared globular in shape with highly basophilic cytoplasm, large, spherical and central nuclei with non-condensed chromatin, and many peripheral nucleoli of different sizes; a monolayer of follicular cells surrounded diplotene cells (Fig. 1c). In 38% of samples, the cortex was mostly occupied by numerous and large diplotene oocytes whereas patches of proliferation were restricted in the periphery of the ovary (stage IX); in the remaining 6% ovaries were underdeveloped (Fig. 1d and Table 1).

**Table 1:** Frequency of stages of ovarian development in one week recent metamorphs of *H*. *intermedia*.

Group	Ovary stage (%)		
	Underdeveloped^a^	Stage VIII^b^	Stage IX^c^
Control (n = 16)	6	56	38
5 μg/L (n = 14)	50^***^	35.7	14.3
50 μg/L (n = 14)	21.4^**^	57.2	21.4

^a^Underdeveloped ovary were determined by the presence of large areas of proliferation (numerous oogonia and nests of leptotene-pachytene oocytes) and none and/or few early diplotene oocytes.

^b,c^Categorization of ovarian developmental stages following the criteria described by Ogielska and Kotusz^36^.

Sample size and frequency are reported. Asterisks indicate statistically significant differences between treatments when compared to the control using the two-tailed Fisher’s exact test (^***^p < 0.001 and ^**^p < 0.01).

Testes were bilateral compact organs and, by stereomicroscope, it was possible to note the granular appearance and the absence of lobulations; left testis was longer and larger than right one (Fig. 1e).

Based on histological observations, in the males examined (stage VIII *sensu* ^37^), testes displayed the formation of the typical arrangement in seminiferous tubules (Fig. 1f–h). Each seminiferous tubule had a germ tissue with primary spermatogonia and few secondary spermatogonia (Fig. 1g,h). Primary spermatogonia were large cells with irregular aspect, voluminous eosinophilic cytoplasm and a highly polymorphic nucleus; these germ cells appeared singly located adjacent to the basal lamina of the seminiferous tubules (Fig. 1g,h). Early secondary spermatogonia were recognizable within developing membranous cyst; they were smaller, with a more pronounced colour and less lobulated nuclei than primary spermatogonia (Fig. 1g,h). Darkly stained and flattened somatic cells (Sertoli cells) were located in the central part of testis where they will develop in so-called *rete testis* (Fig. 1f,g).

#### Sex ratio

Based on both gross morphology and histology, the observed phenotypic sex ratio of the control juveniles was 53% females and 47% males, which did not significantly differ from the 50:50 (female:male) sex ratio expected for amphibians (Table 2).

**Table 2:** Summary table of evaluated endpoints.

Group	n^a^	Metamorphic success (n)^b^	Days to complete metamorphosis^b^	n^c^	% Females^d^	% Males^e^	Sex ratio (♀:♂)	GMCI^f^	LSI^g^
Control	80	69	58.5 (0.2)	30	53	47	1:0.9	3.29 (0.13)	3.37 (0.14)
5 μg/L	80	45	62.3 (0.9)	26	54	46	1:0.9	3.12 (0.15)	3.46 (0.16)
50 μg/L	80	35	63.2 (1)	22	64	36	1:0.6	3.34 (0.12)	3.69 (0.20)

^a^Initial sample size.

^b^Data are modified from Bernabò *et al*.^26^.

^c^Number of one week recent metamorphs of *H*. *intermedia* used for histological observations and phenotypic sexed.

^d^Percentage of phenotypic females.

^e^Percentage of phenotypic males.

^f^GMCI = gonad-mesonephros complex index.

^g^LSI = liver somatic index.

Values are reported as means (±standard error).

#### Kidney morphology

**Figure 2:**
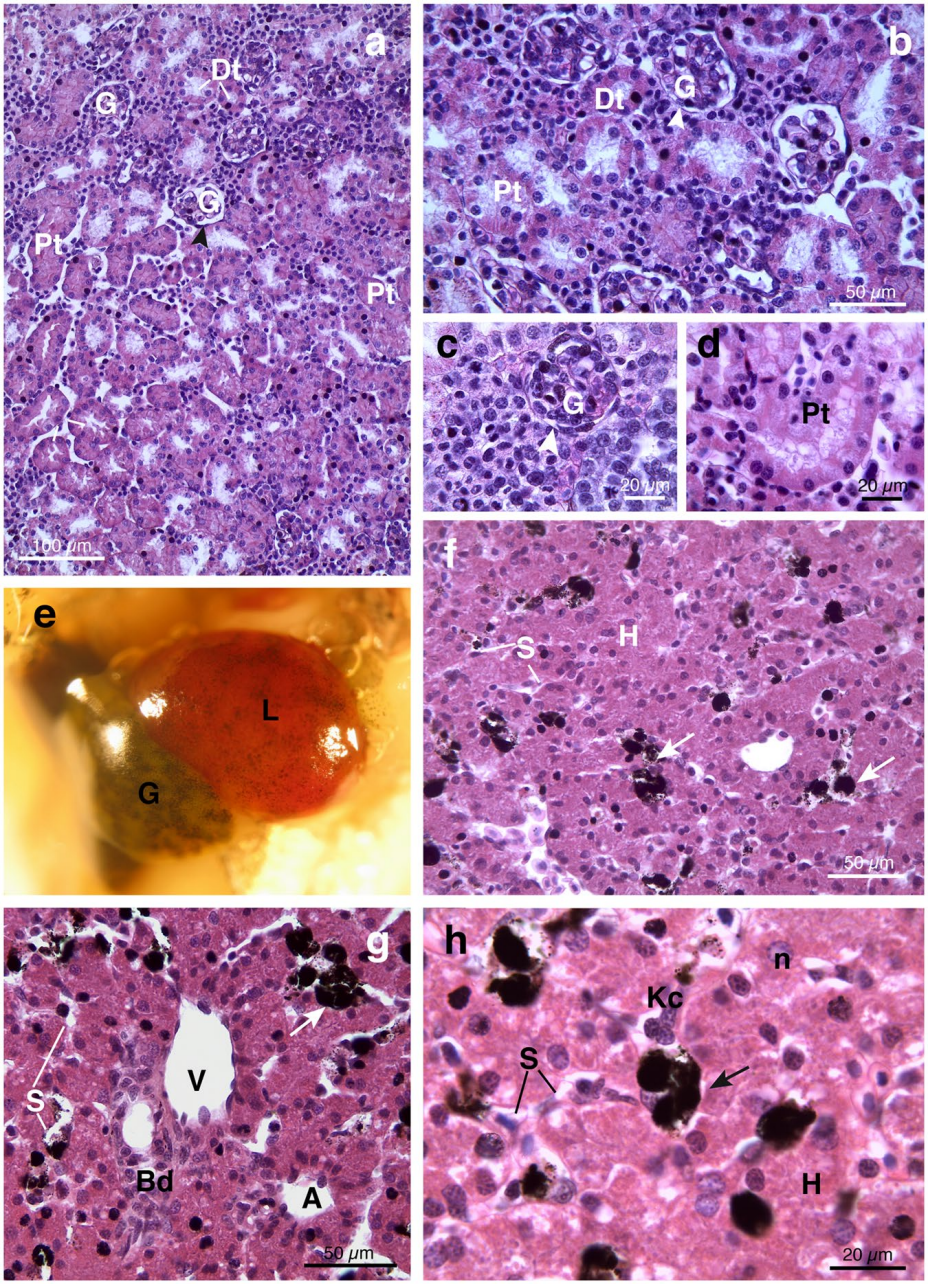
Representative photomicrographs and histological sections of kidney and liver from *Hyla intermedia* juveniles under basal condition. (**a**,**b**) Renal parenchyma showing typical architecture with filtration unit, the capsule of Bowman surrounding the glomerulus, and renal tubules. (**c**) Particular of renal corpuscles formed by a globular mass of specialized capillaries, enclosed into the Bowman’s capsule. (**d**) Note the luminal brush borders of the proximal tubules. (G = glomerulus; Dt = distal tubule; Pt = proximal tubule; arrowheads = Bowman’s capsule). (**e**) External view of liver showing an orange-brown color and pigmented cells. A large dark-green gallbladder is also visible. (**f**) Histological section showing the homogenous arrangement of hepatic tissue. (**g**) Hepatic portal area constituted by bile duct, branches of both portal vein and hepatic artery. (**h**) Particular at higher magnification showing large hepatocytes with rounded nuclei and granulated cytoplasm; Kupffer cells are anchored to sinusoid endothelium. (L = liver; G = gallbladder; H = hepatocytes; S = sinusoid; V = branch of the portal vein; A = branch of the hepatic artery; B = bile duct; Kc = Kupffer cells; n = nucleus; arrows = melanomacrophages complex). All cross-sections are H&E.

The gross morphology of the kidneys resembled the definitive adult mesonephros. The kidneys were paired structures highly vascularized in close contact with the gonads; each kidney showed an elongated oval shape with narrow apical portions and a wider median part (Fig. 1a,e). By light microscope (LM), it was possible to distinguish renal corpuscles and distal tubules distributed in the ventromedial zone of renal parenchyma whereas proximal tubules and collecting tubules were located in the dorsal portion of the kidney (Fig. 2a). Renal corpuscles showed complete Bowman’s capsule and well organized central glomeruli composed of capillary loops (Fig. 2a–c). The renal tubule was divided into the neck segment, the proximal tubule, the intermediate segment and the distal tubule, which opens into the collecting tubule (Fig. 2a). The proximal tubule was made by cuboidal, granulated epithelial cells with acidophilic cytoplasm and was characterized by a dense brush border at luminal surface (Fig. 2b,d). The distal tubule was composed of simple cuboidal epithelium (Fig. 2a,b).

#### Liver morphology

*H*. *intermedia* liver showed an orange-brown pigmented coloration with black spots due to the melanomacrophages (Fig. 2e). The hepatic parenchyma had a compact appearance and was formed by hepatocytes arranged in clusters and cords; sinusoids of different size were interspersed within hepatocytes (Fig. 2f–h). A wide population of large melanin-containing cells (i.e. melanomacrophages) was distributed close or within sinusoids (Fig. 2f–h). Macrophages (or Kupffer cells) could also be recognized (Fig. 2h). In the hepatic portal area it was possible to note branches of the portal vein and hepatic artery along with bile duct composed by simple cuboidal epithelium (Fig. 2g). Hepatocytes were polyhedric in shape with round nuclei and a clear, granulated cytoplasm (Fig. 2h).

### Pyrimethanil exposed groups

#### Sex ratio and gonadal morphology

Based on gross morphology and successive histological validation, phenotypic sex ratio was not significantly different from an equal sex ratio (χ^2^ = 3.019, *df* = 2, p = 0.221) (Table 2) in all experimental groups. Histological analysis also revealed that none of the exposed animals showed sex reversal or abnormal gonadal intersex. Furthermore, exposure to fungicide did not cause significant differences in gonad-mesonephros complex index (GMCI) (One-Way ANOVA, F_2,79_ = 0.7185, p = 0.4906) compared to control group.

**Figure 3:**
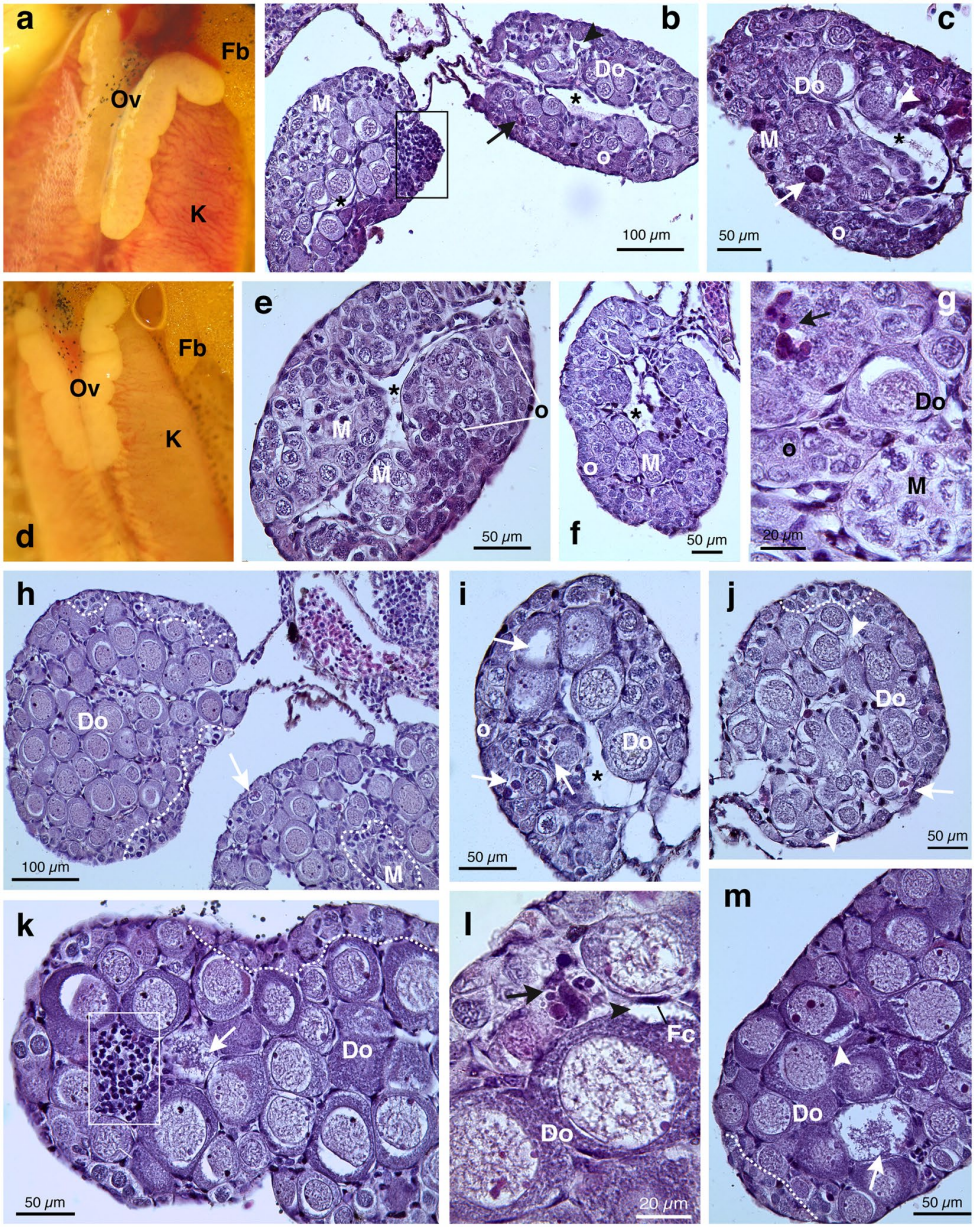
Representative photomicrographs and histological sections of phenotypic females of *H*. *intermedia* juveniles after exposure to pyrimethanil. Ovaries from animals exposed to 5 µg/L (**a**–**c**,**f**–**h**) and 50 µg/L (**d**,**i**–**m**). (**a**,**d**) Exposed animals maintaining a typical ovarian gross morphology. (**b**,**c**,**e**–**g**,**i**) Histological section of exposed animals showing a poorly developed ovarian structure; the cortex is filled with oogonia or early oocytes. Ovarian cavity lined by somatic cells is still distinguishable (*) (**h**,**j**–**m**) Ovaries at stage VIII or IX. The ovarian tissue is composed by growing previtellogenic diplotene oocytes; external patches of dividing oogonia and/or nests of leptotene-pachytene oocytes (marked by dotted lines) could be seen. (**b**,**c**,**h**–**m**) Diffuse degeneration phenomena affecting all germ cell types (arrows). (**b**,**c**,**j**,**l**,**m**) Note the enlargement of intercellular spaces and the detachment of diplotene oocytes from the follicular cell (arrowheads) and (**b**,**k**) mononuclear cells infiltration (marked by rectangle). (**g**,**l**) At higher magnification the presence of numerous apoptotic bodies frequently engulfed by macrophages is clearly detected (arrows). (Ov = ovaries; K = kidney, Fb = fat body; Do = diplotene oocytes; Fc = follicular cells; o = oogonia; M = meiocytes at leptotene-pachytene stages). All cross-sections are H&E.

During dissection under stereomicroscope, it was possible to note that gross morphology of females from pyrimethanil exposed groups was maintained and all individuals showed well-developed ovaries with typical external lobulation (Fig. 3a,d). On the contrary, the LM analysis revealed severe abnormalities in both oogenesis progression and histological features (Table 1 and Fig. 3b,c,e–m). In fact, despite typical ovarian structure with presence of the central cavity, underdevelopment of the meiotic oocytes nests was detected. The ovarian tissue was mostly filled by oogonia and nests of early meiotic prophase oocytes and none or few newly formed diplotene oocytes were observed (Fig. 3b,c,e–g,i).

Statistical analysis of gonad differentiation, revealed that this delay was significant for both pyrimethanil exposed groups (χ^2^ = 56.75, df = 4, p < 0.0001). In detail underdevelopment was recognized in the 50% of ovaries from the low concentration group and in the 21.4% from the high one (Fisher’s exact test, ^***^p < 0.001 and ^**^p < 0.01, respectively) (Table 1).

Moreover, ovarian tissue was affected by several types of alterations, such as conspicuous degeneration phenomena involving all germ cell types (Fig. 3b,c,h–m), large presence of macrophages and apoptotic bodies (Fig. 3g,l); enlargement of intercellular spaces and detachment of diplotene oocytes from the enveloping follicular cells (Fig. 3b,c,j,l,m), and diffuse mononuclear cell infiltration (Fig. 3b,k).

**Figure 4:**
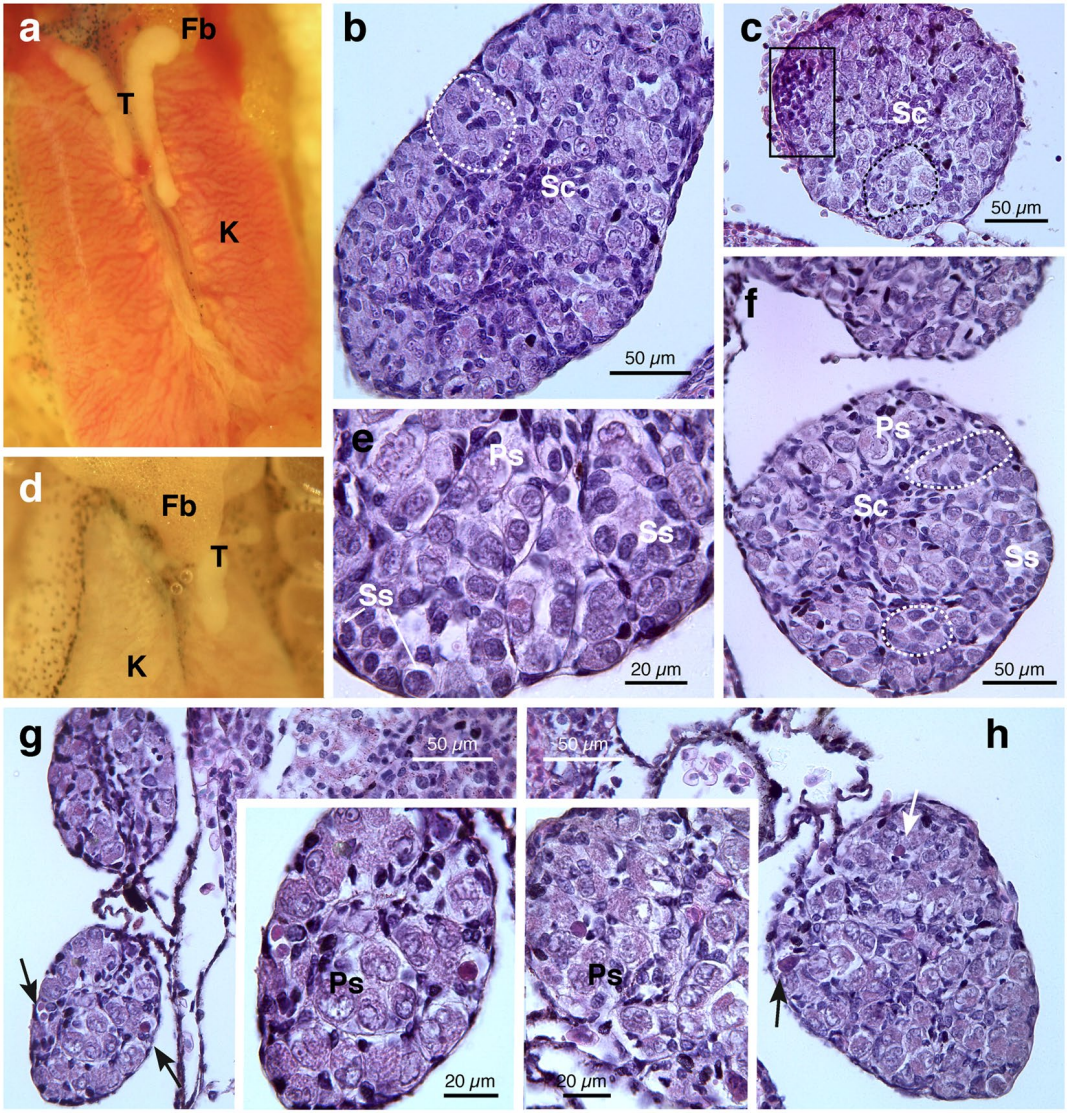
Representative photomicrographs and histological sections of phenotypic males of *H*. *intermedia* juveniles after exposure to pyrimethanil. Testes from animals exposed to 5 µg/L (**a**–**d**) and 50 µg/L (**e**–**h**). (**a**) Testicular normal gross appearance in exposed animal. (**b**,**e**,**f**) Cross-sections showing well preserved testicular tissue with primary and secondary spermatogonia within seminiferous tubules (marked by dotted lines). (**c**) Note the presence of mononuclear cells infiltration (marked by rectangle). (**d**) Macroscopic ventral view of a specimen with only one left testis with reduced dimension. (**g**,**h**) In some treated animals seminiferous tubules tend to be less organized and alterations of testicular tissue are detected: including enhancement of the intercellular spaces, primary spermatogonia/germ cells showed hydropic tumefaction or signs of focal necrosis (arrows). Details of interest are magnified in the inset. (T = testes; K = kidney, Fb = fat body; Sc = somatic cells; Ps = primary spermatogonia; Ss = secondary spermatogonia). All sections are H&E.

Males from pyrimethanil exposed groups showed no significant differences in testes gross morphology compared to control (Fig. 4a). However, in low concentration group we found two individuals with only one testis (the right one was absent) (Fig. 4d), unfortunately we did not obtain the histology and therefore these were exclude from successive analysis; these animals exhibited edema at metamorphosis (see below). In samples of both concentrations groups, most of individuals displayed a well preserved histology and the testes appeared organized in seminiferous tubules (Fig. 4b,c) with big primary spermatogonia and several cysts of rounded secondary spermatogonia (Fig. 4e,f). However in some samples from both experimental groups, alterations of testicular tissue have been observed: seminiferous tubules tended to be less well organized with an enhancement of the intercellular spaces, primary spermatogonia/germ cells showed signs of degeneration; mononuclear cell infiltrates were frequently detected (Fig. 4g,h).

#### Kidney histopathology

**Figure 5:**
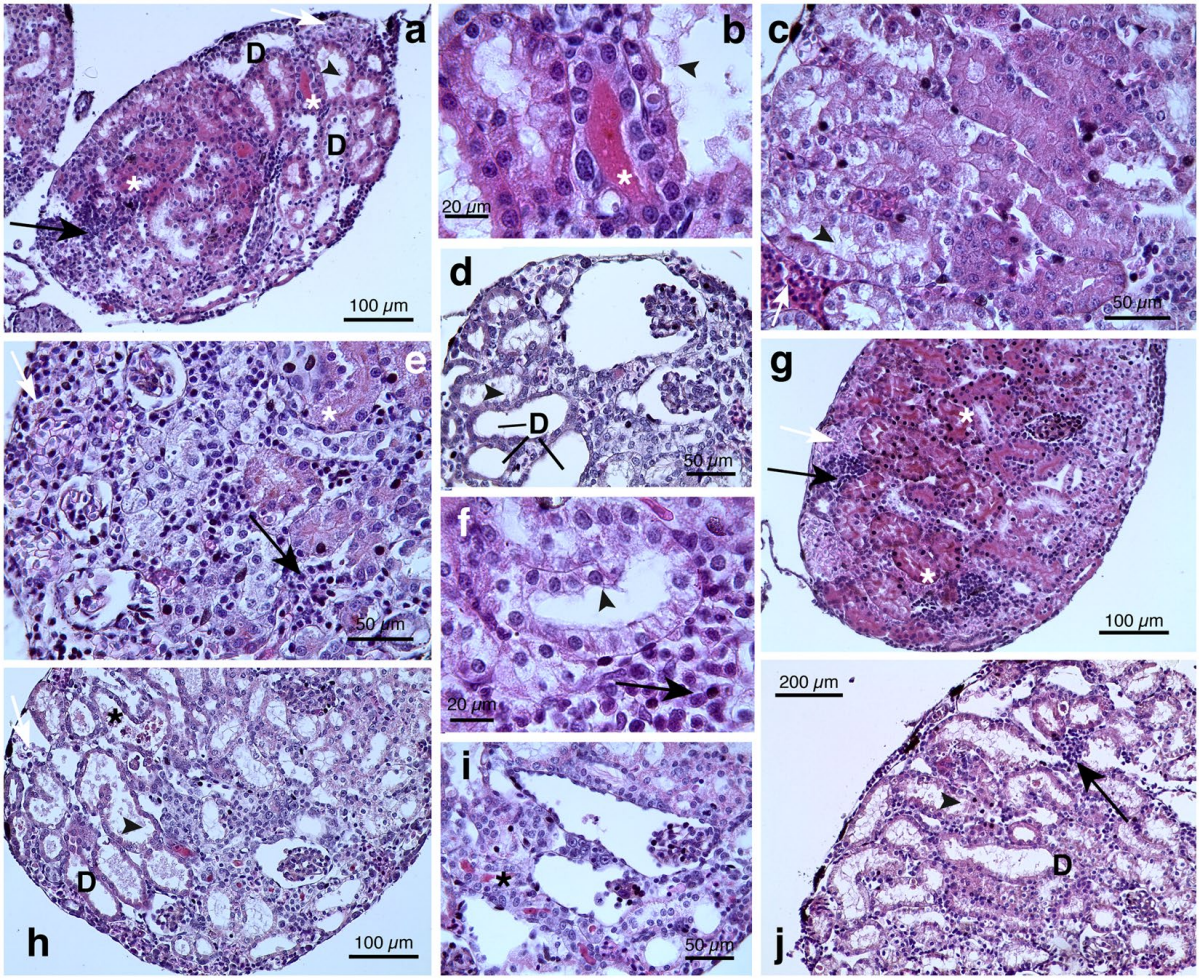
Histopathological examination of kidney tissue in *H*. *intermedia* juveniles after chronic exposure to pyrimethanil. Representative light micrographs showing marked kidney impairments in *H*. *intermedia* after exposure to 5 µg/L (**a**–**f**) and 50 µg/L (**h**–**j**) of pyrimethanil. Extensive signs of tubular degeneration are detected including: (**a**,**d**,**h**,**j**) renal tubular dilation (D); (**b**,**f**) destruction of brush border (arrowheads) clearly appreciable at higher magnification; (**b**,**c**,**e**,**f**,**h**–**j**) diffuse loss of the integrity of the cells, hydropic swelling, vacuolization and apoptotic cells. (**a**,**b**,**g**–**i**) The renal tubules contain intraluminar proteinaceous deposits (*) that appear as dark pink eosinophilic material. **(d**,**e**,**h**–**j**) Considerable glomerular shrinkage and enlargement of Bowman’s capsula can be observed. (**a**,**c**,**e**–**h**,**j**) Inflammatory phenomena are frequently detected: infiltration of inflammatory cells, mainly mononuclear cells (black arrows); haemorrhage (white arrows). All cross-sections are H&E.

Histological analysis revealed many morphological changes in renal parenchyma of animals exposed to both fungicide concentrations (Fig. 5), despite gross morphology was preserved (Fig. 4a). Intensity of impairment varied depending on individual response, but the regular arrangement of kidney was always impaired by a general disorganisation of tissue and consistent histologic features of necrosis. In all samples, we observed some pathological effects in both renal tubules and corpuscles. One of the most frequent alteration were the inflammatory response, associated with infiltration of both macrophages and mononuclear cell (Fig. 5a,e–g,j), the presence of haemorrhage (Fig. 5a,c,e,g,h,j), and apoptotic bodies. Renal tubules displayed severe damages such as a pervasive tubular dilation (Fig. 5a,d,h,j), hydropic swelling, with or without cytoplasmic vacuolization, and a general loss of cytoplasmic detail (pale-staining and poor cytoplasm of necrotic cells) (Fig. 5). The presence of proteinaceous fluid within the renal tubules (characterized by homogenous dark pink material) was frequently observed (Fig. 5a,b,g–i). Moreover, at higher magnification was evident the destruction of brush border in the proximal renal tubule (Fig. 5b,f). A marked widening of Bowman spaces and shrinkage of the capillaries were observed (Fig. 5d,e,h–j).

#### Liver histopathology

Exposure to fungicide did not cause significant differences in liver somatic index (LSI) (Kruskal-Wallis test, p = 0.3584) compared to control group.

**Figure 6:**
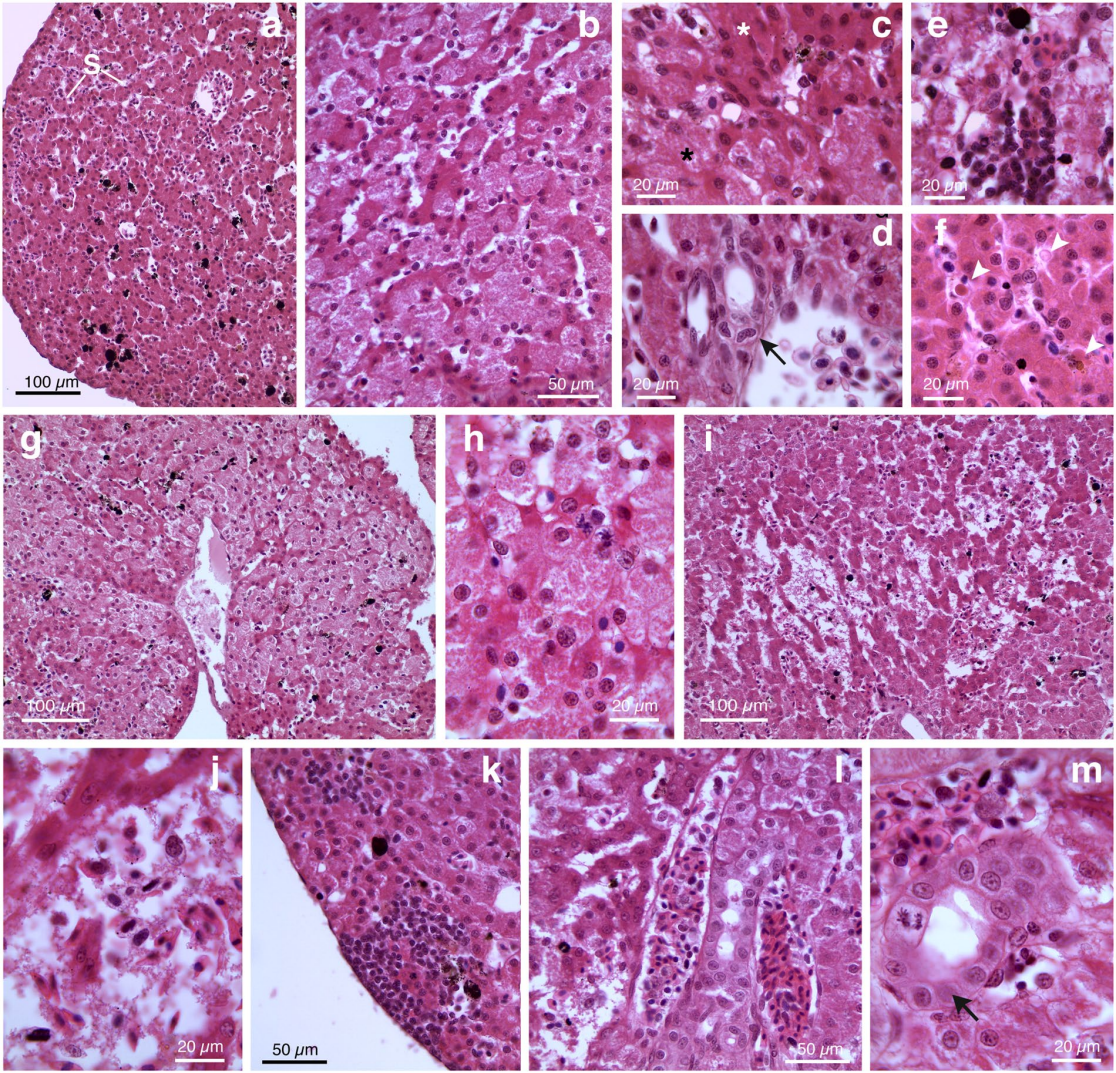
Histopathological examination of liver tissue in *H*. *intermedia* juveniles after chronic exposure to pyrimethanil. (**a**–**f**) Representative light micrographs showing alterations in *H*. *intermedia* liver after exposure to 5 µg/L of pyrimethanil. (**a**) Congestion and dilatation of sinusoids (S). (**b**) Liver parenchyma dyschromia. (**c**) Necrotic cells with pale cytoplasm (white asterisk) and apoptotic cells (black asterisk) with a hypereosinophilic cytoplasm. (**d**) Degenerating bile ducts (arrow). (**e**) Mononuclear cells infiltration. (**f**) Apoptotic cells (arrowheads). (**g**–**m**) Representative light micrographs showing the alterations observed in *H*. *intermedia* liver after exposure to 50 µg/L of pyrimethanil. (**g**) Note the diffuse and extensive degenerative phenomena in liver parenchyma. (**h**) Particular at higher magnification of hepatocytes in degeneration. (**i**,**j**) Hepatic parenchyma completely disorganized in which is possible to note extended necrotic and hemorrhagic zones. (**k**) Mononuclear cell infiltration. (**l**) Blood vessels congestion. (**m**) Note the disorganization in bile ducts (arrow). All cross-sections are H&E.

Histological examinations revealed, in liver of animals exposed to pyrimethanil, considerable morphological changes (Fig. 6). In samples from the low concentration group, the liver appeared highly vascularized and the numerous blood vessels appeared often occluded (Fig. 6a). The large amount of blood resulted in sinusoidal congestion and dilation giving to the liver a loose appearance (Fig. 6a). In addition, the liver parenchyma dyschromia, due to degenerative phenomena, was evident at higher magnification (Fig. 6b). In some areas, hepatocytes showed a clear, foamy cytoplasm whereas in other cases the cytoplasm appeared highly hypereosinophilic (Fig. 6c). In the portal area, also cuboidal cells forming interlobular bile ducts displayed signs of degeneration (Fig. 6d). Signs of inflammation (e.g. infiltration of mononuclear cells) in sinusoids and among hepatocytes were frequently detected (Fig. 6e). In addition, a large amount of apoptotic bodies could be seen (Fig. 6f).

In samples from the high concentration group, the intensity of degenerative phenomena greatly increased (Fig. 6g–m). The hepatic architecture was completely lost and diffuse degeneration along with hepatic dyschromia was observed in all samples (Fig. 6g). Both hypereosinophilic and clear degenerating hepatocytes could be detected (Fig. 6h). In large areas, massive or sub-massive necrosis was observed (Fig. 6i) and it was possible to note haemorrhage and cellular debris (Fig. 6j). Mononuclear cell infiltration (Fig. 6k) and vessels congestion (Fig. 6l) were conspicuous. Bile ducts disorganization was observed in portal area (Fig. 6m).

#### Immunofluorescence

**Figure 7:**
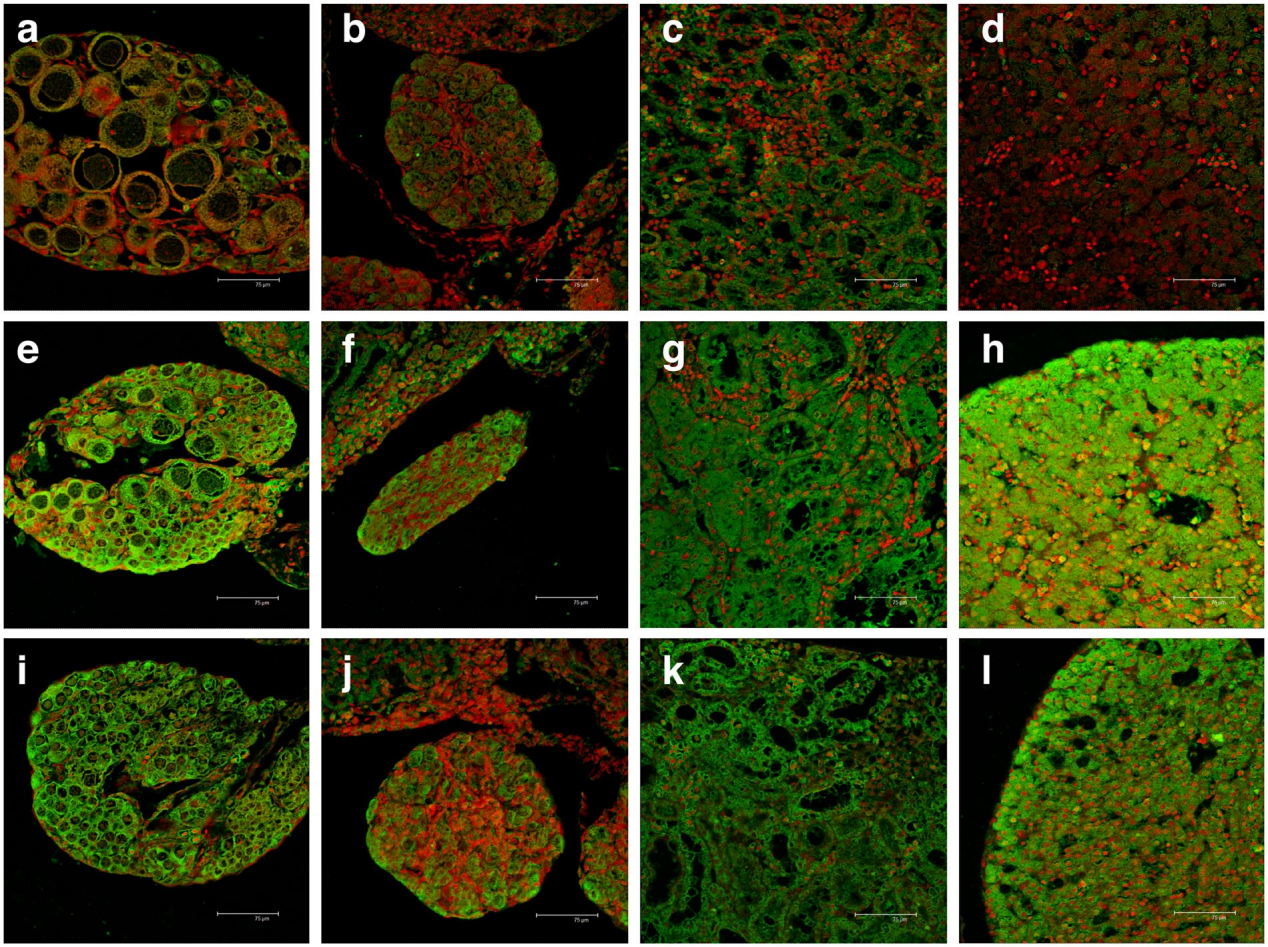
Immunolocalisation of caspase 3. Confocal micrograph of *H*. *intermedia* ovaries (**a**,**e**,**i**), testes (**b**,**f**,**j**), kidney (**c**,**g**,**k**) and liver (**d**,**h**,**l**) sections labeled with rabbit polyclonal antibody against caspase-3 (green – FITC labeled); nuclei are labeled with propidium iodide (red). (**a**–**d**) In control specimens no or extremely weak immunoreaction could be revealed. (**e**–**l**) After exposure to both pyrimethanil concentrations, it was possible to note a marked increase in caspase-3 immunoreactivity in samples from both low (**e**–**h**) and high (**i**–**l**) concentration groups.

Caspase-3 immunodetection revealed no labeling or an extremely weak one in all tissues from control group (Fig. 7a–d). After exposure to both pyrimethanil concentrations, it was possible to note a marked increase in Caspase-3 immunoreactivity (Fig. 7e–l).

The increase of staining is tissue-specific and both ovaries (Fig. 7e,i) and liver (Fig. 7h,l) exhibited the most intense caspase positivity. We detected a dose-related enhancement of signal in kidney (Fig. 7g,k) and testes (Fig. 7f,j), whereas in liver (Fig. 7h) and ovaries (Fig. 7e) the peak of expression has been observed in samples from the low concentration group. In both ovaries and testes, the signal was particularly evident at the periphery of the organs but all gonadal tissue was stained (Fig. 7e,f,i,j). Similarly, in the liver the signal was mainly detected at peripheral region of the organ (Fig. 7h,l). On the contrary, in kidney the signal was uniform through the section (Fig. 7g,k).

## Discussion

Our results were successful in demonstrating that the anilinopyrimidine fungicide pyrimethanil has the potential to induce histological alterations in tissues of the Italian tree frog *H*. *intermedia*. We revealed that a long term exposure (78 days) to environmentally realistic concentrations of this fungicide caused severe histopathologic damages in gonads, liver and kidney. To the best of our knowledge, this is the first report describing the effects of pyrimethanil on the morphology of these organs in amphibian. The findings of this study are consistent with our previous results showing the reduced survival rate, the alteration in development and metamorphic traits and the incidence of severe malformations induced by this fungicide in the same species. Moreover, we described for the first time the histological features of *H*. *intermedia* gonads, liver and kidney, under basal condition.

Histological observations on *H*. *intermedia* juveniles, under basal conditions, revealed that both ovaries and testes exhibit a general arrangement similar to that described for *H*. *arborea* and for other species belonging to Hylidae family^36–38^.

Anuran gonads are most susceptible to malformation during the period of sexual differentiation and the time in which this takes place has been demonstrated to differ among species^11, 36, 39, 40^. After exposure to pyrimethanil, we detected an unmodified phenotypic sex ratio in both experimental groups; also morphological analysis revealed no sex reversal or abnormal gonadal intersex at metamorphosis thus supporting the hypothesis that this fungicide is not able to induce feminization or masculinization in *H*. *intermedia*. Numerous studies in amphibians indicate the incidence of intersex as the main gonadal abnormality induced by exposure to EDCs^9, 11, 12, 41^. According to previous results on the effects induced by EDCs on amphibian sexual development, the susceptibility and the incidence of sex reversals are a species-specific response^42^ and references therein. Our results emphasize the importance of studies on non-model species when evaluating the effects of EDCs and other pollutants, since an extrapolation of single-species data to other may be misleading^41^.

On the other hand, we clearly showed that a chronic exposure to pyrimethanil causes severe abnormalities in both oogenesis progression and histological features of gonads, despite the well maintained gross morphology. The presence of unpaired gonad observed in two males from the low concentration group has been previously reported by Goldberg^38^ in both males and females of *Scinax fuscovarius*, another species belonging to Hylidae, in natural condition. Aside, the unique gonad in male from our experiments, displayed a normal appearance thus suggesting that no correlation exists between the incidence of this anomaly and the exposure to xenobiotic.

The role of histological evaluation is largely recognized as the key tool in phenotypic sex determination^12, 36^. Our results strongly support the concept that histological analysis is able to disclose a host of alterations, beyond intersex incidence, that may underpin reproductive pathologies, also when anatomical organization is largely unaffected. One of the most important findings of our study is the highly significant underdevelopment of the meiotic oocytes since it clearly indicates that female fertility may be compromised by pyrimethanil, even at very low concentrations. As pyrimethanil interferes with normal sexual differentiation, causing a delay in gonads development, its endocrine disruption potential is evident and largely supported by this study. A similar effect, although much more pronounced, has been reported in juveniles of *Rhinella arenarum* after exposure throughout embryo-larval development to 0.25 and 2 mg/L of the formulated fungicide Maxim^®^ XL (a mixture of two active ingredients: Fludioxonil and Metalaxyl-M)^43^.

There is surprisingly few data on the effects of fungicides on reproductive system, even though they are known or presumed to act as EDCs. Reproductive function in amphibians may be impaired through multiple mechanisms acting individually and/or synergically. As for other pesticides, aryl hydrocarbon receptor (AhR)-mediated effects have been hypothesized for pyrimethanil in studies *in vitro* ^33^. The AhR is a transcriptional factor belonging to the basic helix-loop-helix (bHLH)-Per-ARNT-Sim (PAS) family that mediates a wide range of biological and toxicological responses in several different species and tissues.

It has been suggested that AhR-mediated cellular responses may have a pivotal role in female reproductive process^44^. A growing body of evidence established the interactions between the AhR and different intracellular pathways after exposure to a variety of synthetic and naturally occurring chemicals^45^. Whether the exact molecular events is determined or not, xenobiotic compounds that are able to modulate endogenous endocrine messengers inevitably impair reproduction and developmental events^44^.

In addition, we detected a number of histological abnormalities in both testes and ovaries after exposure to pyrimethanil. Both apoptosis and degeneration considerably increased in exposed groups suggesting the coexistence of different pathological pathways. We revealed an intense labeling for caspase-3, a key mediator of apoptosis, in gonads of both males and females exposed to pyrimethanil and in particular the most intense immunoreactivity has been observed in females from the low concentration group. Programmed cell death in the reproductive systems intervenes removing damaged cells^46^ thus explaining in part the reduction of gonadal maturation induced by pyrimethanil. In mammal’s oocyte, apoptosis is regulated by concentrations of circulating sex steroids with estrogens preventing atresia and androgens increasing this process^47^. Mackenzie and colleagues^18^ suggested that the testosterone increase, due to the treatment with an aromatase inhibitor, may be the cause of the ovarian atresia observed in two species of Ranidae. The role of steroids in amphibian’s gonad differentiation has not definitely been clarified and available information on the steroid hormonal control of gonad differentiation is scarce and conflicting^42, 48^. However, one may speculate that aromatase inhibition would be one of the mechanisms involved in gonadal impairment after exposure to pyrimidine fungicides^49, 50^. The AhR-mediated action does not exclude other alternative physiological responses and in fact pyrimethanil also induce inflammation as demonstrated by the presence of macrophage and mononuclear cellular infiltrate.

Our observations on healthy *H*. *intermedia* liver confirm the general organization described for other amphibian species, and other vertebrates^51, 52^. The hepatic parenchyma is composed of hepatocytes, arranged in clusters and cords, surrounding a network of sinusoids. We showed the presence of a well defined portal triad, thus contributing to the discussion on whether a portal triad would be a common structural feature of vertebrate liver or not^51, 52^.

For their crucial role in detoxification of most xenobiotic compounds, the liver is considered as the main target organ for the assessment of histopathological changes. Here we clearly demonstrated that a chronic exposure to pyrimethanil resulted in modifications of liver morphology in a dose related manner. The dilation and congestion of blood vessels and sinusoid were one of the most evident results of pyrimethanil application. Similar findings were reported in several amphibian species after exposure to both pesticides and heavy metals^34, 53, 54^. Some authors suggested that endothelial cells alterations may affect the blood flow from the hepatic artery and veins to the central vein thus resulting in extravasations of red blood cell and sinusoids dilation^53, 55^. We also showed a remarkable liver tissue dyschromia highlighting the establishment of different pathologic response in the same tissue. Numerous interrelated biochemical and molecular mechanism are involved in cellular outcomes to pollutants; proliferative status, repair enzyme capacity, and the ability to induce proteins promoting/inhibiting cell death determine the cellular fate. It is now becoming increasingly clear that both necrosis and apoptosis can occur in response to the same type of insult^56, 57^.

After exposure to pyrimethanil, a common alteration observed in *H*. *intermedia* hepatocytes was the appearance of numerous apoptotic bodies. Programmed cell death is an important mechanism for the removal of senescent or damaged cells, and it has been demonstrated that apoptosis is induced by several EDCs *in vitro* studies^58^. Moreover, it has been reported that in fish liver other xenobiotics exert their toxicity via apoptosis^59^. In our experiment the apoptosis occurred in conjunction with the necrosis thus demonstrating the attempt of liver tissue to restore homeostasis, and the otherwise strong cytotoxic effect of pyrimethanil. As confirmed by immunodetection of caspase-3, this process peaked in the hepatocyte of low concentration groups. Probably the high concentration may favour the onset of the degenerative phenomena.

The necrotic cells were easily recognizable in *H*. *intermedia* liver by their loose pale cytoplasm. The swelling of cellular bodies and nuclei led to the accumulation of cellular debris into the cytosol and interstitial space and to the subsequent recruitment of inflammatory cells. Vacuolization and necrosis in hepatic cells and haemorrhage have been reported in *B*. *variabilis* after exposure to the insecticide carbaryl^34^ and in both fish and amphibians after exposure to copper^60, 61^. Morphology and histology of *H*. *intermedia* kidney under basal conditions correspond to that described for other amphibian species^62, 63^. As in other vertebrates, amphibian kidney plays a major role in maintaining a stable internal milieu and it is well acknowledged as an important target for xenobiotic. We showed that, despite a preserved gross morphology, pyrimethanil is able to affects kidney histology in *H*. *intermedia* since all individuals from exposed groups displayed some alterations, involving both renal tubules and corpuscles. The most severe effects were observed in samples from the high concentration group, although the intensity of injury varied, to some extent, depending on the individual response.

Histological alterations frequently detected were a considerable infiltration of inflammatory cells, a pervasive tubular dilation, a widening of Bowman spaces and shrinkage of the capillaries. Both the inflammatory cells infiltration and the dilation of renal parenchyma can be induced by different types of toxic injury to the tubule epithelium^64^. The destruction of brush border observed in the proximal renal tubules of *H*. *intermedia* has also been observed in amphibians after exposure to heavy metals and the authors suggested that this may explain the well known alteration of both reabsorptive and secretory functions of proximal tubules induced by metals^64, 65^. Other changes, often observed, were hydropic swelling, presence of proteinaceous fluid within the renal tubules, and occurrence of both necrotic and apoptotic cells.

Limited studies are available for comparison with our results to evaluate the consequences of pesticides on the amphibian kidney but our findings are consistent with the few previous reports on this organ. Comparable pathological effects have also been reported in frogs inhabiting contaminated area^35^ and after exposure to several heavy metals^64, 66^. Similar changes were reported in adult of *B*. *variabilis* after carbaryl oral administration^34^ and in adult of *Pelophylax ridibundus* after intraperitoneal injections of both the copper-containing fungicide Champion 50WP^67^ and the pyrethroid insecticide Talstar 10EC^54^.

It seems that different xenobiotic may induce similar pathological effects on kidney also independently from the administration route. Nevertheless, the high sensitivity of the kidney to pollutants indicates the histopathological evaluation of this organ as a good biomarker for risk assessment studies.

On the basis of our data, it is conceivable that the noxious effect of pyrimethanil can result from several pathological processes affecting different key compartments of amphibian. In our opinion, when evaluating the harmful potential of a xenobiotic it is important to conduct a broad and in-depth study through a multi-organ evaluation in order to disclose all the putative and subtle pathological effects. Moreover our results strongly support the essential role of cross-check data from both laboratory and field studies, when assessing a site-specific risk. In fact, according to our earlier results, when such severe multi-organ alterations are detected in amphibian from an apparently uncontaminated site, a previous contamination event cannot be excluded.

## Conclusion

This is the first evidence of the effects induced by pyrimethanil on gonads, liver and kidney histology and point out the hazardous properties of this commonly used fungicide for non-target species. Current pesticides risk assessment procedures for amphibians, depend on surrogate species. At the moment, the EFSA is developing a Guidance Document for amphibians and reptiles risk assessment. The information presented in this paper, enhancing our understanding of the potential impacts of fungicides on amphibians, would contribute to develop better environmental management practices and appropriate measures for the amphibian protection.

## Methods

All experimental procedures for animal handling and tissue removal were undertaken in compliance with approved Animal Care and Use Committee and were approved by the Italian State Office of Environment. Rome, Italy. Permit number: PNM-2011-0002086. All animals not used in experiments were released in the place of collection.

The experimental design for animals exposure to pyrimethanil has been described in detail in Bernabò and colleagues^26^. We report here a concise description.

### Study species

*H*. *intermedia* is a native small tree frog, which is located still quite abundant throughout Italy; it is listed on Appendix II of the Bern Convention and Annex IV of the EU Habitats Directive. Although Italian tree frog is not in danger of extinction (IUCN), this endemic species is potentially threatened by local habitat loss to urbanization, climate change and water pollution (presumably by agrochemicals)^68^.

As for other species of the family Hylidae, sexual differentiation of gonad takes place in early larval development and equal numbers of distinct male and female gonadal phenotypes are expected without an intersex developmental phase^36, 37^. Sex is determined by sex chromosomes (male heterogamety)^69^.

This species was selected because it typically breeds in a variety of lentic environments, frequently located in modified habitats such in agricultural field or urban areas. Furthermore, *H*. *intermedia* constitutes an interesting case study due to the lack of data on the patterns of gonadal differentiation and on its susceptibility to EDCs.

### Collection and animal husbandry

Three newly laid (up to 3 d) egg clutches were collected from natural unpolluted ponds (Calabria, Southern Italy; 39°21′36″N 16°9′3″E; elevation 387 m a.s.l.) during the spring of 2013. In the laboratory, eggs and larvae were acclimated in 40 L aerated glass aquaria (60 cm × 35 cm × 30 cm) filled with aged tap water renewed twice a week.

### Experimental setup and exposure conditions

The nominal concentrations of pyrimethanil used here were: 5 μg/L and 50 μg/L, referred as the low and the high respectively. Pyrimethanil (purity 99.9%, Cas No: 53112-28-0) was purchased from Sigma Aldrich Chemie GmbH (Steinheim, Germany). Doses selection, range finding test, preparation of solutions, water parameters measurements, and chemical analysis was based on our previous paper^26^. The analyses confirmed the accuracy of the stocks and dilutions, and that water fungicide concentrations were fairly constant over the course of a week.

Experiments started when tadpoles reached the developmental Gosner^70^ stage 25 (feeding and free-swimming tadpoles). Tadpoles were chronically exposed via a 7-day static renewal exposure regimen from Gosner stage 25 to 46 (end of metamorphosis and complete tail resorption). The exposure period lasted 78 days and the whole experiment was conducted in quadruplicate. For each experimental unit, twenty tadpoles of comparable body dimension were randomly chosen and assigned to 15 L aerated glass tanks (40 cm × 25 cm × 20 cm) containing the appropriate test solution. Throughout the study period, animals were held under a natural light:dark photoperiod (at 22 ± 1 °C, median pH 7.3) and fed every three days with boiled organic spinach *ad libitum*. Food waste and debris were removed daily using a fine mesh net.

At the initiation of at least one forelimb emergence (Gosner stage 42), individuals were housed into semi-aquatic tanks containing a thin layer of respective treatment solution (0.5 L) and dry areas (stones and soil) to complete metamorphosis. They were not fed throughout this period, since anuran larvae typically do not feed for several days during metamorphosis (due to remodelling of mouth and digestive organs).

Mortality and/or completion of tail resorption, in each experimental unit, were monitored daily. Immediately after metamorphosis, the froglets were transferred in plastic terrariums with a moist substrate, water in shallow Petri dishes, and fed *ad libitum* with *Drosophila melanogaster*. At this point the individual recognition was performed and each animal was given a unique identification number; time to initiate and complete metamorphosis, length, and weight for each frog were recorded^26^.

### Morphofunctional analysis

One week after completion of metamorphosis, froglets were deeply anesthetized by immersion in 0.1% tricaine methanesulfonate (MS-222, Sigma-Aldrich Chemicals Co., St. Louis, MO). A subset of the surviving juveniles from each experimental unit was utilized for histological analysis (Control n = 30, 5 μg /L n = 28, and 50 μg/L n = 22). Gross morphology evaluation and phenotypic sex determination were performed under a stereomicroscope (Leica MZ APO, Leica Microsystems, Wetzlar, Germany equipped with Canon camera) using a drop of Bouins’ solution to enhance coloration.

Liver and gonad-mesonephros complex were quickly excised. Each organ was weighed in an analytical precision scale (0.001 g) (MettlerBas Bal 300) for calculation of liver somatic index (LSI) and gonad-mesonephros complex index (GMCI) as follows: LSI = (liver weight/body weight) × 100; GMCI = (gonad-mesonephros complex weight/body weight) × 100.

Samples were placed in Bouins’ liquid for 24 h at 4 °C and then dehydrated in an increasing series of ethanol, cleared in xylene and embedded in paraffin wax. Sections (7 μm) were serially cut and mounted on positive charged slides. For liver, step sections were taken at 50 μm intervals until the maximum diameter of the samples was attained. For gonad-mesonephros complex, serial sections from the dorsal, middle, and ventral region were taken.

Before evaluation, slides were randomized and coded, and all histological sections were blindly screened to validate phenotypic sex, identify morphological abnormalities, and analyse the progression of gonadal maturation and immunoresponse.

#### Light microscope analysis

Sections for morphological analysis were stained with hematoxylin and eosin (Panreac, Barcelona, Spain), and photographed by light microscope equipped with a digital camera (Leica DME, ICC50 HD, Leica Microsystems, Wetzlar, Germany). Analyses of ovaries and testes, and staging of gonadal development were performed according to ref. 36 and ref. 37. Froglets were defined as females based on the presence of an ovarian cavity and oogonial cells and/or nests of oocytes (early meiotic and/or diplotene); animals were defined as male based on the presence of a large medullary region and of a distinct testicular structure, with spermatogonia into early seminiferous tubules.

In order to determine *H*. *intermedia* gonad differentiation pattern (i.e. differentiated, semi-differentiated or undifferentiated), individuals raised apart were also analysed at selected developmental Gosner stages (27, 30, 37, and 42).

#### Immunofluorescence

Dewaxed sections were rinsed with distilled water and phosphate buffer (PBS) and then incubated for 30 min in a moist chamber with 20% normal serum to block non-specific sites. Unwashed sections were incubated overnight at 4 °C with a rabbit polyclonal antibody to caspase-3 (diluted 1:100 – Sigma-Aldrich Chemical Co., St. Louis, MO, USA). After several washes in PBS, sections were incubated with fluorescein isothiocyanate-conjugated γ-globulins sheep anti-rabbit (diluted 1:100 - Sigma-Aldrich Chemical Co., St. Louis, MO, USA) for 30 min at room temperature. Finally, slides were counterstained with propidium iodide (1:200 - Sigma-Aldrich Chemical Co., St. Louis, MO, USA), which binds to ribonucleic acid and labels cell nuclei.

To examine non-specific binding (negative control) primary antibody was substituted with non-immune normal serum. The sections were observed under a Leica TCS SP2 confocal laser scanning microscope (LSM).

### Data analysis

Analyses of data were performed using Graph Pad Prism 5.00 (GraphPad Software Inc., San Diego, CA, USA), at significance level of 0.05. As reported in Bernabò *et al*.^26^, no tank effect was detected and data from replicates per treatment groups were pooled into one data set. Assumptions of normality and homoscedasticity were tested with D’Agostino & Pearson omnibus and Bartlett’s tests, respectively. Nonparametric tests were used if data could not be transformed to meet the assumptions for analysis of variance (ANOVA).

To test whether sex ratio in each group deviated from the expected 50:50 (female:male), χ^2^ test was performed. For evaluations of frequency of ovarian developmental stages, we first used χ^2^ test; if differences (exact p ≤ 0.05) were found, Fisher’s exact tests (2-sided) were applied for pairwise comparisons between control and exposure groups with respect to frequency of underdeveloped versus developed ovary.

To test the effect of pyrimethanil exposure on LSI, Kruskal-Wallis test followed by Dunn’s Multiple Comparison Test was performed. Significant differences in GMCI between fungicide exposed and control animals were tested using a One-Way ANOVA followed by Bonferroni’s Multiple Comparison Test.

## Electronic supplementary material

Hyla intermedia gonads differentiation
