# Supplementary material for: Chronic exposures to fungicide pyrimethanil: multi-organ effects on Italian tree frog (Hyla intermedia)
Source: Sci Rep. 2017 Jul 31;7:6869. doi: 10.1038/s41598-017-07367-6 (PMC5537256; doi:10.1038/s41598-017-07367-6)
Supplement: Supplementary file 1 — Hyla intermedia gonads differentiation [file 41598_2017_7367_MOESM1_ESM.pdf]

Chronic exposures to fungicide pyrimethanil: multi-organ effects on Italian tree frog (*Hyla intermedia*)

Ilaria Bernabò, Antonello Guardia, Rachele Macirella, Sandro Tripepi, Elvira Brunelli

Hyla intermedia gonads differentiation

Histological analysis revealed that by Gosner[36] stages 27-30 (n=5) ovary differentiation has begun. The first event was the reduction of the medulla and the appearance of a central lumen lined by newly formed epithelial cells. In the cortical region it was possible to distinguish proliferating primordial germ cells and primary oogonia. By stage 30 (n=5), in developing testes undifferentiated somatic cell populated the medullary region and primary spermatogonia were visible; a central lumen was not observed (see also stage 42).

We demonstrated that in *H. intermedia*, during pre-metamorphic larval stages, developing gonads directly differentiated into ovaries or testes, and the gonadal differentiation is completed by the end of metamorphosis (sexually distinct male and female gonads without an intersexual phase).

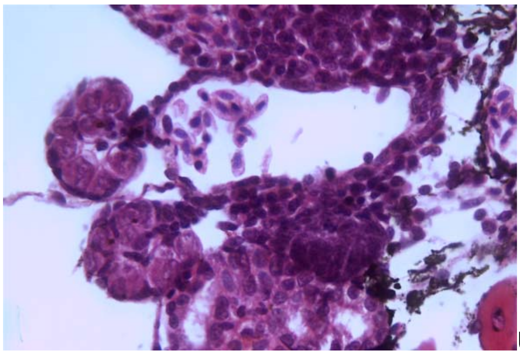
Undifferentiated gonad (stage 26)

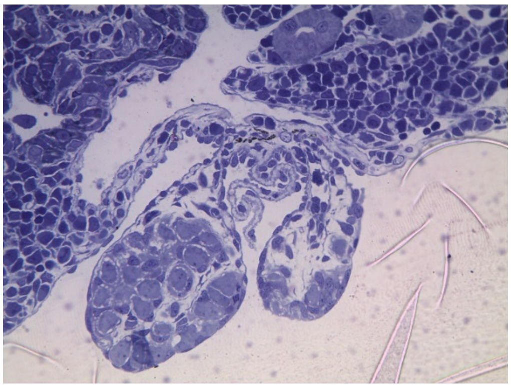
Differentiating female (stage 30)

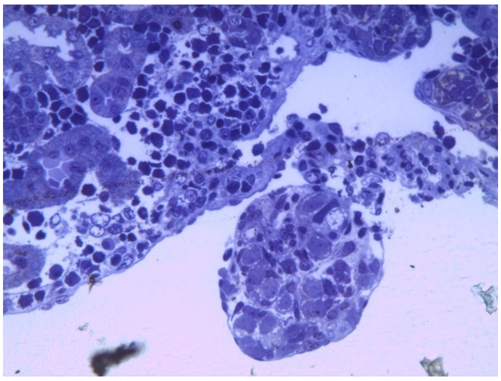
Differentiating male (stage 30)

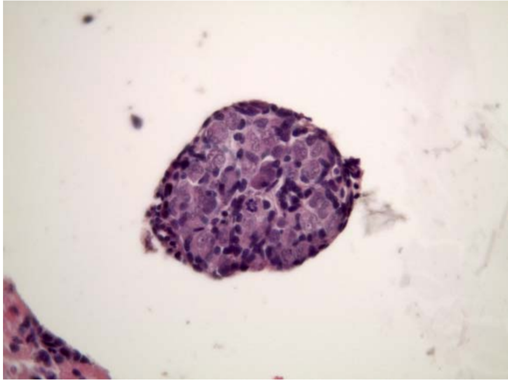
Differentiating male (stage 42)
